# Community perceptions on the factors in the social food environment that influence dietary behaviour in cities of Kenya and Ghana: a Photovoice study

**DOI:** 10.1017/S1368980022002270

**Published:** 2023-03

**Authors:** Milkah N Wanjohi, Rebecca Pradeilles, Gershim Asiki, Michelle Holdsworth, Elizabeth W Kimani-Murage, Stella K Muthuri, Ana Irache, Amos Laar, Francis Zotor, Akua Tandoh, Senam Klomegah, Fiona Graham, Hibbah Araba Osei-Kwasi, Mark A Green, Nathaniel Coleman, Kobby Mensah, Robert Akparibo, Richmond Aryeteey, Emily K Rousham, Nicolas Bricas, Marco Bohr, Paula Griffiths

**Affiliations:** 1 Maternal and Child Wellbeing Unit, African Population and Health Research Center, P.O Box 01787-00100, Nairobi, Kenya; 2 School of Sport, Exercise and Health Sciences, Loughborough University, Leicestershire, Loughborough, UK; 3 Health and Systems for Health Unit, African Population and Health Research Center, Nairobi, Kenya; 4 UMR MoISA (Montpellier Interdisciplinary Centre on Sustainable Agri-food systems), (Université Montpellier, CIRAD, CIHEAM-IAMM, INRAE, Institute Agro, IRD), Montpellier, France; 5 Population Dynamics and Reproductive Health Unit, African Population and Health Research Center, Nairobi, Kenya; 6 Warwick Center for Applied Health Research and Delivery, Warwick Medical School, University of Warwick, Coventry, UK; 7 Department of Population, Family and Reproductive Health, School of Public Health, University of Ghana, Legon, Accra, Ghana; 8 Department of Family and Community Health, School of Public Health, University of Health and Allied Sciences, Ho, Volta Region, Ghana; 9 Population Health Sciences Institute, Faculty of Medical Sciences, Newcastle University, Newcastle upon Tyne, UK; 10 Department of Geography, University of Sheffield, Sheffield, UK; 11 Department of Geography & Planning, University of Liverpool, Liverpool, UK; 12 Department of Obstetrics and Gynaecology, University of Ghana Medical School, Korle Bu, Accra, Ghana; 13 Department of Marketing and Entrepreneurship, University of Ghana Business School, Legon, Accra, Ghana; 14 School of Health and Related Research, The University of Sheffield, Sheffield, UK; 15 French Agricultural Research Centre for International Development (CIRAD), Montpellier Cedex 5, France; 16 School of Art and Design, Nottingham Trent University, Nottingham, UK

**Keywords:** Social factors, Food environment, Photovoice, Dietary behaviour, Urban, Africa

## Abstract

**Objective:**

To explore communities’ perspectives on the factors in the social food environment that influence dietary behaviours in African cities.

**Design:**

A qualitative study using participatory photography (Photovoice). Participants took and discussed photographs representing factors in the social food environment that influence their dietary behaviours. Follow-up in-depth interviews allowed participants to tell the ‘stories’ of their photographs. Thematic analysis was conducted, using data-driven and theory-driven (based on the socio-ecological model) approaches.

**Setting:**

Three low-income areas of Nairobi (*n* 48) in Kenya and Accra (*n* 62) and Ho (*n* 32) in Ghana.

**Participants:**

Adolescents and adults, male and female aged ≥13 years.

**Results:**

The ‘people’ who were most commonly reported as influencers of dietary behaviours within the social food environment included family members, friends, health workers and food vendors. They mainly influenced food purchase, preparation and consumption, through (1) considerations for family members’ food preferences, (2) considerations for family members’ health and nutrition needs, (3) social support by family and friends, (4) provision of nutritional advice and modelling food behaviour by parents and health professionals, (5) food vendors’ services and social qualities.

**Conclusions:**

The family presents an opportunity for promoting healthy dietary behaviours among family members. Peer groups could be harnessed to promote healthy dietary behaviours among adolescents and youth. Empowering food vendors to provide healthier and safer food options could enhance healthier food sourcing, purchasing and consumption in African low-income urban communities.

Globally, Africa is among the regions with the highest rate of urban population increase; about 60 % of the population is projected to live in urban areas by 2050^(1)^. Kenya and Ghana exemplify these trends. By 2020, about a third (28 %) and slightly more than half (57 %) of the Kenya and Ghana’s population respectively was urban^(2)^. Rapid and unplanned urbanisation in low- and middle-income countries is associated with urban poverty and various emerging environmental and health hazards^(3)^. It is also linked to changes in social economic and physical food environments and a subsequent nutrition transition^(4)^ characterised by shifts in people’s food habits such as an increase in the consumption of unhealthy foods that are high in calories, fat, salt and sugar^(4)^. Unhealthy diets are estimated to make a greater contribution to the non-communicable disease burden than alcohol, smoking and physical inactivity, combined^(5)^.

Both Kenya and Ghana are experiencing a nutrition transition, and an increasing trend in overweight and obesity^(6)^. Between 2000 and 2016, the prevalence of overweight and obesity combined increased, from 28 % to 45 % and 12 % to 20 % among women and men respectively in Kenya^(7)^. In Ghana, overweight and obesity combined increased, from 38 % to 58 % and 16 % to 27 % among women and men respectively in the same period^(8)^. Studies from these countries indicate a higher prevalence of overweight and obesity in women and urban residents^(9)^. Further evidence indicates a higher rate of increase in overweight and obesity among the poorest population segments in urban Africa^(10)^. A systematic review of dietary behaviours in both countries also revealed relatively low consumption of healthy foods, such as fruit and vegetables (52 %) and widespread consumption of unhealthy foods such as sugar sweetened beverages (40 %)^(11)^.

The social food environment, defined as the food-related interactions between friends, family and peers,^(12)^ has a major influence on individuals’ intent and actual food behaviour^(13)^. The social food environment, including social norms, networks and contexts that promote the adoption of unhealthy dietary behaviour, is a potential underlying factor for the development of obesity^(14)^. Understanding the role of the social food environment in influencing dietary behaviour is important to identify effective interventions for the promotion of healthier diets and prevention of diet-related non-communicable diseases, especially in urban contexts^(15)^. Currently, there is demand for nutrition policies and interventions to be more evidence-based, context and culturally specific, with recommendations for qualitative research to enhance the understanding of social processes that drive dietary behaviours in urban Africa’s social environment^(15)^. However, the specific social influences of dietary behaviours in urban contexts are not well documented in Africa^(16)^, despite the increasing urbanisation and growing trends in overweight/obesity.

Hence, this study aimed to explore the perspectives of communities living in urban cities in Kenya and Ghana on the factors in the social food environment that influence their dietary behaviours. The ‘Photovoice’ methodology that uses the support of photography taken by local people to talk about their environment was used to provide the evidence for this aim^(17)^. Photovoice allows for an emic approach in investigating how local people identify their own food environment and how they perceive it. It also allows the researcher to see issues ‘through the eyes’ of study participants and communities. This methodology has been used in other high- and low-income countries to understand social food environments as perceived by adults^(18)^, as well as adolescents and youth^(19)^.

## Methods

### Study setting

This study was part of a wider project^(20)^ conducted in three rapidly growing urban African cities; Accra and Ho (Ghana) and Nairobi (Kenya). The study in Ho was part of a larger project *(drivers of food choices)* that targeted women of reproductive age only, whereas the studies in Accra and Nairobi were part of the TACLED project, which included both men and women aged 13 to 49 years^(20)^. The different cities represented different contexts in East and West Africa, and different levels of urbanisation and nutrition transition, including major cities (Accra and Nairobi) and a secondary city (Ho).

### Study design

This was a cross-sectional, qualitative study that employed Photovoice methodology. Photovoice is a community-based participatory and visual research methodology in which participants are given a camera to capture conditions and issues in their environment, through photographs^(21)^. The photographs taken then act as a visual prompt for participants, providing them with an opportunity to describe realities, communicate perspectives and raise awareness of complex public health issues in their environments^(17,22)^.

### Sampling and data collection

As this study focused on lower income groups, a list of all deprived neighbourhoods in the selected cities (excluding slums) was compiled. This list was further restricted by retaining neighbourhoods that were deemed to be safe to work in by the research team. One neighbourhood in each city was then randomly selected using a manual lottery method: James Town (Accra), Dome (Ho) and Makadara (Nairobi).

Within the selected neighbourhoods, participants were purposefully recruited using quota sampling based on key characteristics (i.e. age, gender, BMI, socio-economic level, and education level and occupation status) (Supplementary file 1). This was to ensure breadth in the range of views, perspectives and environments that participants were exposed to. The Photovoice study was carried out on a random sub-sample (i.e. a third) of the overall study population of the wider project (target sample: *n* 64 in Accra, *n* 32 in Ho and *n* 48 in Nairobi; total *n* 144). Recruitment took place through the communities, schools and health services. Additional information on the sampling and recruitment strategy can be found elsewhere^(23)^.

### Data collection

The Photovoice activity was conducted between September 2017 and June 2018. The format of the Photovoice prompt and interview guide used for this study was adapted from the conventional format proposed by Wang (1999), to suit the research context. The main adaptation that was made for this project was to conduct one-to-one interviews instead of the more collective workshop or focus group discussion approach that is normally used in Photovoice. The individual approach was used because our initial community engagement activities suggested that most of the targeted participants in urban areas were busy with work or school, and it would be hard to bring them together at the same time. In addition, the safety of group gatherings was considered a problem since Kenya was experiencing political instability at the time of data collection.

Prior to the start of data collection, the Photovoice open-ended interview guide was piloted (Accra (*n* 3), Ho (*n* 3) and Nairobi (*n* 4) and subsequently amended. The amendments mainly included simplifying the guiding questions (Photovoice prompts) and rephrasing of sentences to suit the local contexts. During the period of taking photographs, participants were visited four times in their homes by trained research assistants on a day that was most convenient to them. In the first visit, participants were taken through (i) the consent process, (ii) the Photovoice methodology, (iii) the use of a camera to take different types of photographs and (iv) photo ethics including the *no face or identification details’* protocol to ensure anonymity of people or places. Participants were then requested to take five photographs during one week that best represented (i) a place where you eat food and/or beverage from; (ii) something that makes healthy eating difficult for you; (iii) something that makes healthy eating easy for you; (iv) something that influences what you eat in your (local) area; and (v) a person that influences what you eat in your (local) area. Two follow-up visits were made by research assistants during the week, to check on the progress and address any issues arising with the photography activity.

After one week, follow-up in-depth interviews to discuss the photographs were conducted, in which the participants told the ‘stories’ of the five photographs they had selected and provided a short caption to describe their favourite photograph. The in-depth interviews were conducted by research assistants, using the Photovoice open-ended interview guides (Supplementary file 2). The interviews were conducted mainly in local languages (Swahili for Kenya, & Twi, Ga and Ewe for Ghana) or (less often) in English. The prompts and interviews guides used were translated into local languages by accredited translators, and then back translated into English, to ensure that meaning was not lost. Interviews were digitally recorded and lasted between 45 and 60 min.

### Data analysis and synthesis

Interviews were transcribed verbatim, reviewed for accuracy and coded in NVivo 11 by at least two members of the research team in each study site (MNW/RP/AT/SK/FG/AI). All coders were extensively trained, and double coding of 25 % of the transcripts (*n* 36) was performed to ensure consistency when applying the codebook. Any discrepancies identified during the double coding process were discussed and resolved. External opinion was also sought from another member of our research team (PG) to discuss any unresolved coding approaches.

The approach taken for the development of the codebook and subsequent thematic analysis was both theory-driven, using a priori themes compiled using existing socio-ecological models of dietary behaviours^(12,24)^ and data-driven (grounded), to allow for themes emerging from the data.

The socio-ecological model highlights factors influencing dietary behaviours across four levels: individual (preferences, knowledge, socio-demographic characteristics); social (family, friends, and peers); physical (the home, workplace, schools, restaurants, supermarkets) and macro (food marketing, food production, distribution systems)^(12)^. All the interviews were coded for individual level factors, social environment factors, physical food environment factors and macro-level factors. This manuscript reports a synthesis of the themes and subthemes on factors in the social food environment that are perceived to influence dietary behaviours in the three cities (Accra, Ho and Nairobi). The findings on the role of the individual- and physical-level food environments on dietary behaviours have been published elsewhere^(23)^.

## Results

A total of 142 participants from Nairobi (*n* 48), Accra (*n* 62) and Ho (*n* 32) participated in this study, slightly lower than the targeted sample of 144 participants. Overall, 68·3 % of participants were female and nearly half were 19–49 years old. With regard to participants’ occupation, 35·2 % were in work, 13·4 % in education and 51·4 % not in work nor education. The proportion of participants with a BMI ≥ 25 kg/m^2^ was higher in Kenya (60·4 %) than in Ghana (Accra: 48·4 % and Ho: 46·9 %) (Table 1).

**Table 1:** Socio-demographic characteristics of the participants

	Accra (*n* 62)		Ho (*n* 32)		Nairobi (*n* 48)	
	*n*	%	*n*	%	*n*	%
Gender						
Females	40	64·5	32	100·0	25	52·1
Males	22	35·5	0	0·0	23	47·9
Age						
13–18 years	20	32·3	12	37·5	15	31·3
19–49 years	27	43·5	20	62·5	17	35·4
≥50 years	15	24·2	0	0	16	33·3
Socio-economic status						
Lowest	32	51·6	16	50·0	29	60·4
Low to middle	30	48·4	16	50·0	19	39·6
Occupation						
In work	22	35·5	12	37·5	16	33·3
In education	8	12·9	4	12·5	7	14·6
Not in work or education	32	51·6	16	50·0	25	52·1
BMI						
<25 kg/m^2^	32	51·6	17	53·1	19	39·6
≥25 kg/m^2^	30	48·4	15	46·9	29	60·4

The themes emerging on the influence of social environment on the participant’s dietary behaviour included (1) family members’ food preferences, (2) family members’ health and nutrition needs, (3) social support by family and friends, (4) provision of nutritional advice and modelling food behaviour by parents and health professionals, and (5) food vendors’ services and social qualities.

### Family members’ food preferences

Food preferences for different household members were central to the decisions that participants made on foods purchased, eaten or prepared for the entire household. For instance, in all the three cities, participants acknowledged buying or preparing foods that their children liked or could easily eat or take to school. Among married couples, the food preferences of one spouse influenced their partner’s food choices. Women across all cities reported considering their husbands’ preferences while making decisions on the foods prepared for the family. In Nairobi, some male participants indicated that their food consumption was solely dependent on the foods that their wives cooked for them. Further, some participants from households that had vegetarians or young children reported to mainly cook vegetarian meals or foods that young children could easily eat. Preparing common meals that everyone in the household could eat was seen as convenient, saving time and resources (Table 2).

**Table 2:** Narratives and photographs on the theme ‘household members’ food preferences’

Accra	‘It’s because of my children that I mostly cook at home… At times I ask them what they want to eat and I prepare it and I also eat some of the food. The other day, they wanted to take in ‘Gari soakings’ *(Cassava flour meal)* and I did that for them and I also ate some.’ (Female, 19–49 years, lowest SES, A35).	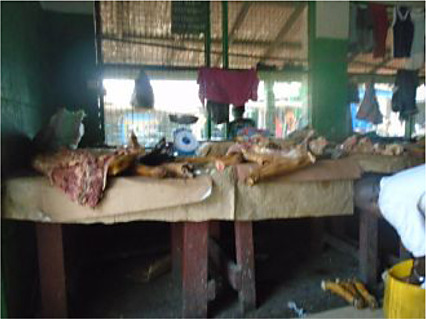
Ho	‘These are my kids. They are the ones that sometimes tell me what we should eat, then I cook it. You can’t just cook whatever you want. Because it is not everyone who will eat what you want to eat. So you will ask, ‘what should we eat?’ and then the kids can say mama, let us eat this or let us cook that’ (Female, 19–49 years, lowest SES, H6).	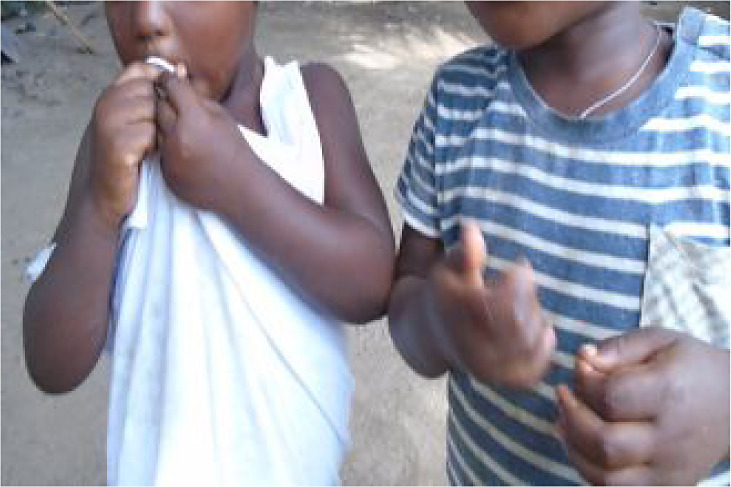
Nairobi	‘These are (my) children eating ‘*Githeri’ (cooked mixture of maize and beans),* it is their favourite, and they make me cook it, every time… In fact, we eat it around 3–4 times a week. This is important… because I value and love them (my children)’ (Female, 19–49 years, low to middle SES, N11).	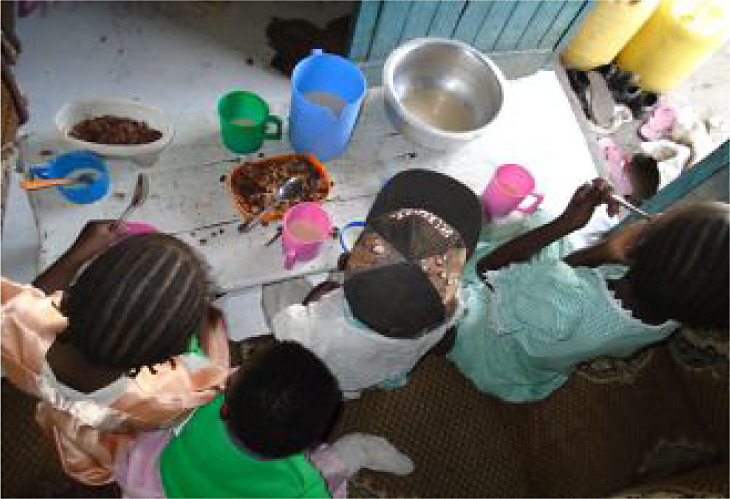
Accra	‘My husband likes fufu *(yam or cassava flour meal)* and soup, banku *(fermented corn and cassava flour meal)* and pepper and fish are his favourite. He loves soupy foods and because of that, I always cook at home, he does not encourage that we buy food from food vendors outside so he always encourage cooking at home’ (Female, 19–49 years, low-middle SES, A34).	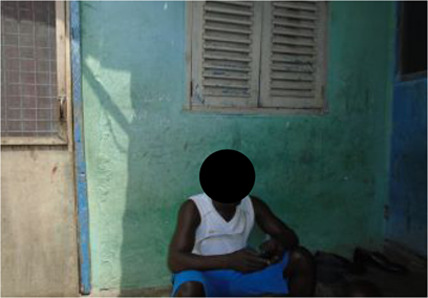
Ho	‘I don’t really like yam, but as for them (husband and children), they like yam, they enjoy every day, so I have to prepare it for them. When I prepare it, he tells me to eat, so I take some and eat. They are the ones who make me eat’ (Female, 19–49 years, lowest SES, H13).	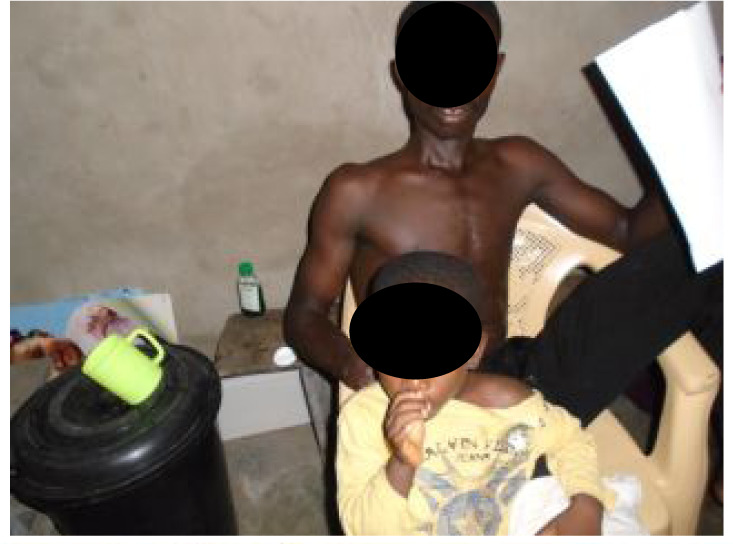
Nairobi	‘Most of the time, I am tired and I don’t have appetite but, my wife makes my eating easier, she influences me to eat because she knows the food that I like and the food that I like is traditional vegetables’ (Male, 19–49 years, low-middle SES, N30). ‘For ‘mrenda’ *(jute mallow)* most of the time I eat because I find that is what my wife has prepared in the house so I cannot leave it since I also like it’ (Male, 50 years and above, lowest SES, N38).	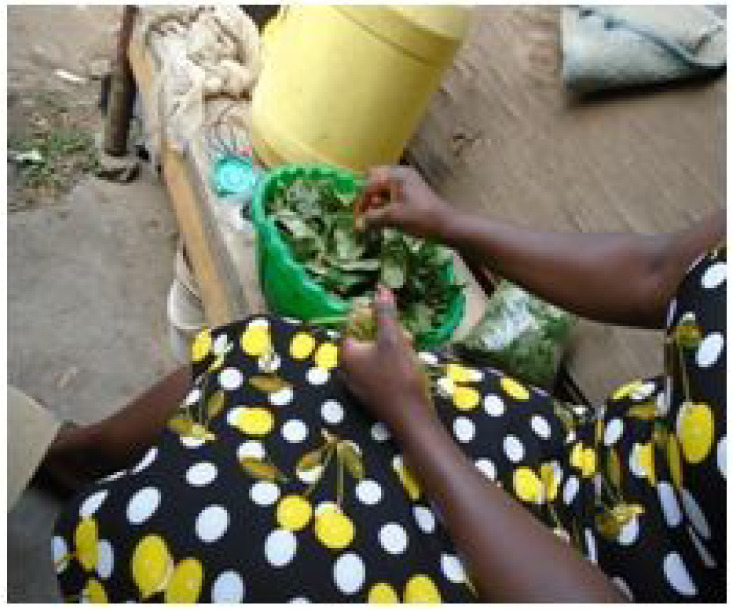

### Health and nutritional needs of family members

Considerations for the health and nutrition of various family members influenced the foods purchased, prepared or eaten at home by the rest of the family. In Ghana (Accra and Ho), female participants, mainly in their role as mothers reported preference for foods perceived to promote their children’s health over those that were considered unhealthy. In Accra (but not Ho and Nairobi), there was a preference for preparing meals at home, citing the reasons that home prepared food is healthier, prevents children from falling sick and is cheaper so children can have enough food to satisfy them (Table 3).

**Table 3:** Narratives and photographs on the theme ‘ health and nutritional needs of the family members’

Accra	‘The banku sold outside will not give you the needed strength and it’s not healthy that is why I prefer to cook at home so my family and children can eat it at home and not fall sick’ (Female, 19–49 years, low to middle SES, A22)	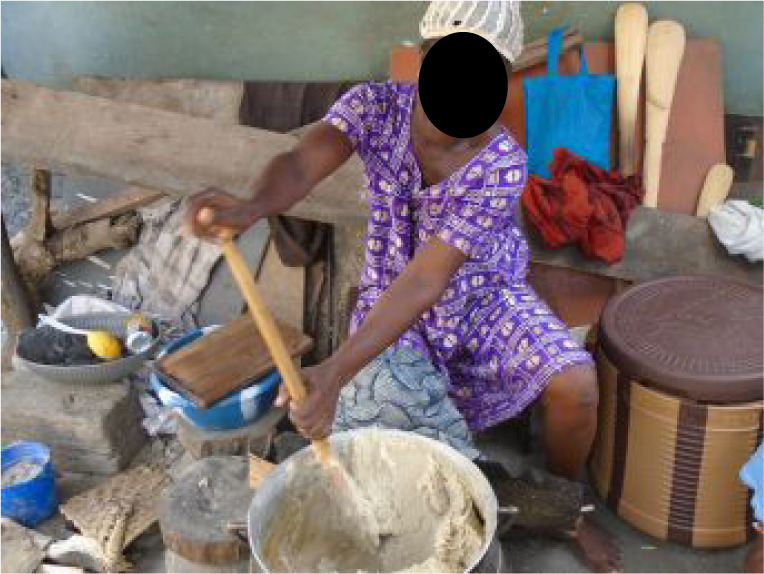
Ho	‘It is because of them (my children) that I eat a lot of healthy foods, so they can get the breast milk to feed on. If I don’t eat properly or healthy, they won’t get enough to feed on. It is because of them that I eat a lot or eat a healthy food to get more breast milk for them to feed on. Because if I don’t eat a lot or eat a healthy food, they will not get the breast milk to feed on and they need to grow well. And if they don’t eat well, me too, I will not get rest’ (Female, 19–49 years, low to middle SES, H57)	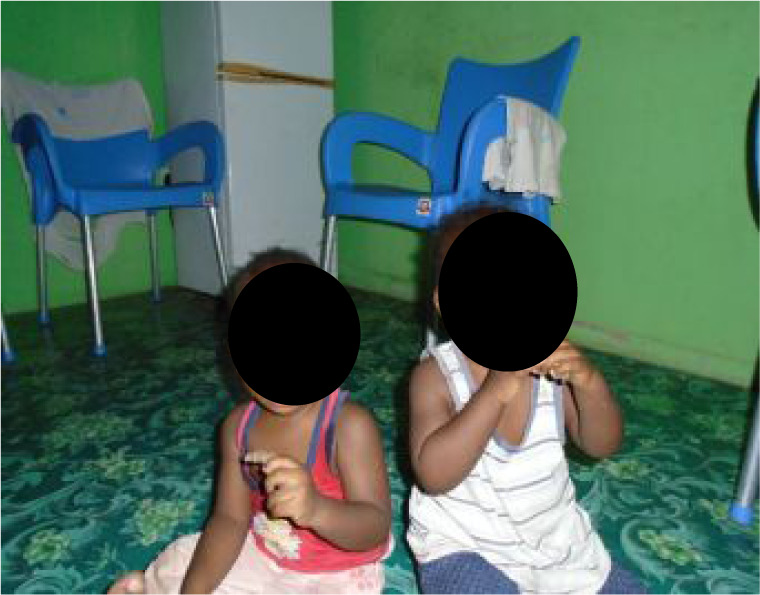

### Social support

Social support, by way of eating together, providing pleasant company during mealtimes and providing support with food provision and preparation emerged as a key influence on participant’s dietary behaviours.

#### Eating together and company during mealtimes

Eating together habitually as a family was regarded as a moment of joy, bonding, fun, an easy and interesting time or a healthy practice. Some participants also reported that eating with family members facilitates ‘eating well’. Further, eating together was seen as an opportunity where family members connect. In Nairobi, breakfast or dinner was the meals that most families reported eating together, while in Accra, eating from the same bowl was a common family practice, but was not commonly mentioned in Ho or Nairobi.

Some of the participants acknowledged that they enjoyed eating in the company of their family as it improved their ‘appetite’ and fostered love and unity in the family

Older participants in all the three cities particularly reported that their children influenced their dietary behaviour by encouraging them to eat and providing pleasant company during mealtimes. Among the younger participants, in Nairobi and Accra, but not Ho, friends were also reported to provide pleasant and enjoyable company during mealtimes (Table 4).

**Table 4:** Narratives and photographs on the sub-theme ‘eating together and company during mealtimes’

Accra	‘We eat together as a family but everyone eats from their own bowl unless we are eating a meal like fufu where we eat from the same bowl. If we are eating a meal like rice and stew, we eat in different bowls. My two sisters normally eat from the same plate but I eat alone and my father and my mother eat in the same bowl’ (Female, 13–18 years, low to middle SES, A45).	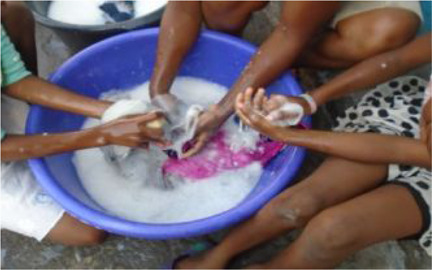
Ho	‘This is my room, I eat from here, but sometimes we eat together, when we eat together I enjoy, than eating alone. I am from an extended family so when they want to give us food, they give it and we all sit together and eat. That is how I grew up, so everyone putting their hands in the food and then we all eat together’ (Female, 19–49 years, low-middles SES, H32).	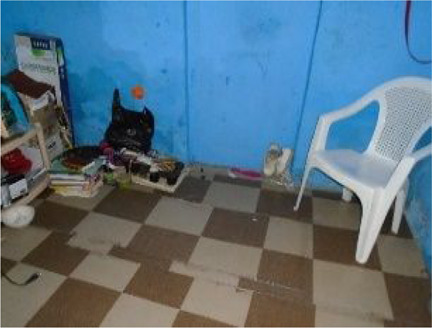
Nairobi	‘We eat together as a family we sit together at the table we eat when we finish we start talking as a family to discuss issues just to enhance the togetherness’ (Male, 13–18 years, lowest SES, N36).	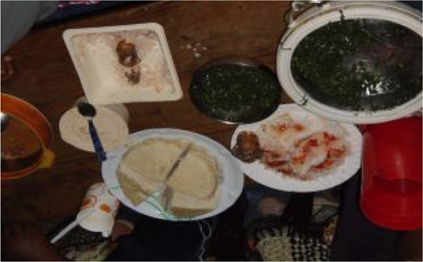
Accra	‘She (my daughter) will say ‘Dad since morning have you taken in food’? Then I will say ‘no’. She will say ‘Dad, don’t do that because you know you have stomach problem’ So she will force me and influence me then I will take the food’ (Male, 50 years and above, low-middle SES, A25).	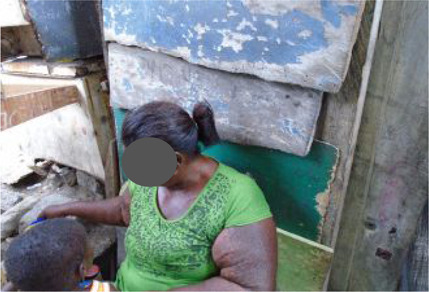
Ho	‘Eating with my kids makes it easy for me to eat. And when we eat together, I enjoy it. When I am eating alone, I am unable to eat well; when we eat together, I am able to eat, they make jokes and make the eating fun, and then we all eat. It is important to eat with your kids and eating together fosters togetherness and friendship and love’ (Female, 19–49 years, low to middles SES, H27).	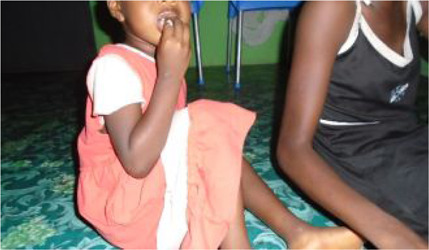
Nairobi	‘Yes, I feel happy because when I eat with her (child) I get the appetite to eat. Because when I eat alone I find it hard to cook because I am alone’ (Female, 50 years and above, lowest SES, N16).	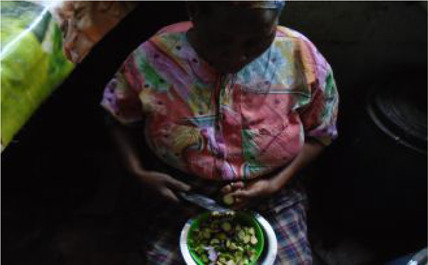
Accra	‘Gbense Friday! A time where me and my friends come together and contribute money to prepare food and eat together… The reason is if we are all eating together we feel happy as compared to if you are eating alone every day. The way the food is, requires that boys come together to eat it’ (Male 19–49 years, low-middle SES, A23)	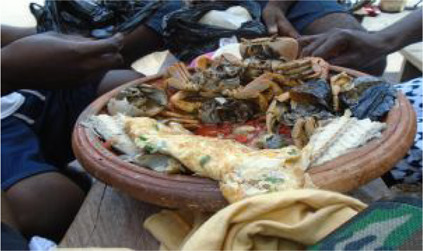
Nairobi	‘I pick this place because most of my friends are there, so if I go there I cannot miss my friends’ (Female 13–18 years, lowest SES, N14).	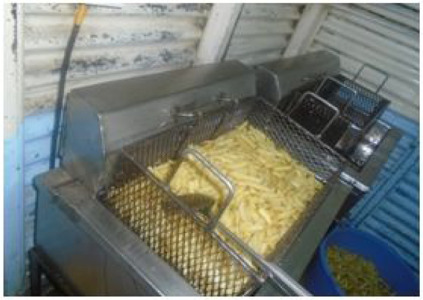

#### Support with food provision and preparation

Husbands were identified as influencers of dietary behaviours, since they provided money for food purchase and, on some occasions, assisted with food preparation. In both Accra and Ho, female participants reported receiving support from their spouses, with food preparation, but this was not reflected in the narratives from Nairobi.

Younger participants in all three cities also highlighted that their friends influenced their dietary behaviours by buying food for them or by lending them money to buy food (Table 5).

**Table 5:** Narratives and photographs on the sub-theme ‘support with food provision and preparation’

Accra	‘My husband is someone who provides the money that I use to buy food stuff and cook for the family to eat. At times I will be preparing banku and we will volunteer to help by frying the fish while I concentrate on preparing the banku… At times he goes to the kitchen to cook for the family whilst I rest. He could prepare the soup and also pound the fufu for us to eat’ (Female, 19–49 years, low-middle SES, A34).	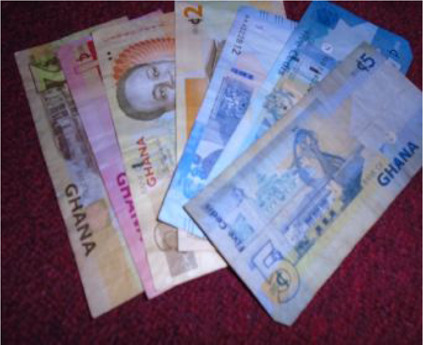
Ho	‘Me, even my husband cooks for me. He can really cook. I don’t have time at all, So the males here also cook. So when you have a wife and you cook for her, it doesn’t mean, she has overcome you’ (Female, 19–49 years, low-middle SES, H15).	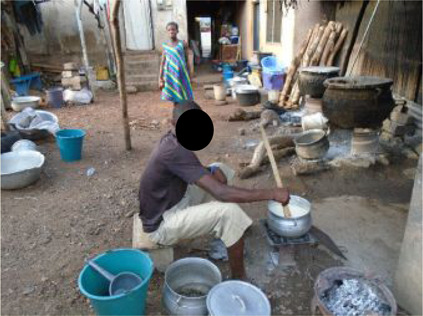
Accra	‘Sometimes he (my friend) has the money so he will be the one to choose the food. Sometimes I also buy and the two of us eat but mostly he is the one having money so most of the times he does and we go and buy food’ (Male 19–49 years, low-middle SES, A23).	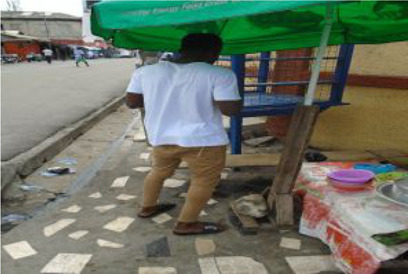
Ho	‘There are times too, when I go and ask my friend to lend me some, and if she also doesn’t have the money, then that days food will be difficult for me and I won’t be able to eat. And I just can’t get up and ask anybody for money, I have to ask from the person I am close with. They are the only ones I can I ask money from, and if they also do not have it, well, then I can’t do anything about it’ (Female, 19–49 years, lowest SES, H13).	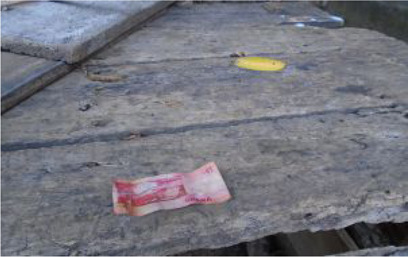
Nairobi	‘This picture is for my friends, you will find that there are times you find yourself you do not have enough money or you do not have money, you will find from one of your friends volunteers to buy you food, if another day also he does not have you are able to sort him out’ (Male, 19 to 49 years, lowest SES, N44).	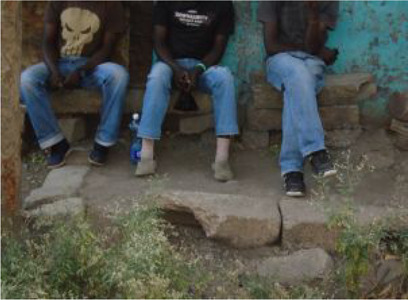

### Nutrition advice and modelling foods behaviours

Parents were commonly mentioned as influencing their children’s food behaviour through providing advice on optimal amounts of food to consume, healthy places to buy food and types of foods to eat and those to avoid, particularly during sickness, pregnancy or lactation. This was more common in Accra and Ho, but less in Nairobi. In addition, parents were reported to model food practices, in Accra and Nairobi (but not Ho), with some participants acknowledging that their preferences of certain foods were based on the foods that they observe their parents eating, preparing, liking or disliking. Some of the younger participants in all the cities further reported that their dietary behaviours were exclusively based on the foods prepared for them by their parents and particularly their mothers, a few reported going along with their parents’ decisions and preferences for family meals even when it was against their own preferences.

Health professionals (nurses, nutritionists and doctors) appeared to influence mainly pregnant women and those with young children, in Nairobi and Accra. This was not reflected in Ho. When visiting health facilities, health professionals advised and encouraged participants to eat certain foods in order to be healthy or promote optimal development for their children. However, a participant in Accra also reported that community nurses advised on food and nutrition targeting children, but not enough for the adult population.

Friends were also mentioned as influencers of younger participant’s dietary behaviour and choices, by advising or encouraging them to eat certain foods (Table 6).

**Table 6:** Narratives and photographs on the theme ‘support with nutrition advice and modelling food behaviours’

Accra	‘She (mother) advises me not to eat too many oily foods and also not to eat a lot of sugar and to eat foods that are hot. She said too much oily food will give me malaria, too much sugar I will get diabetes and it is also not good for my health. She said I should eat heavy food so that I don’t feel dizzy when walking and also to take in palmnut soup so that I will get more blood’ (Female, 19–49 years, lowest SES, A 53).	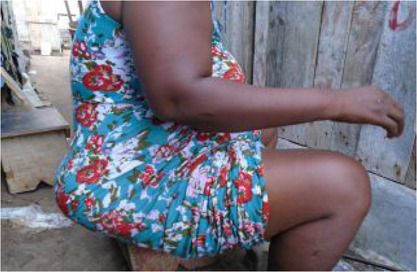
Ho	‘What he (father) does is that, some foods that I want to eat, cause some diseases, since he is older and he has passed through a lot of things in life, he will advise and tell me that it is not good. If I am going to cook such things, he tells me not to cook them. Or he will show me the way I can use them, then he will teach me before I will cook it and eat. And it will not affect my body’ (Female, 19–49 years, low-middle SES, H23).	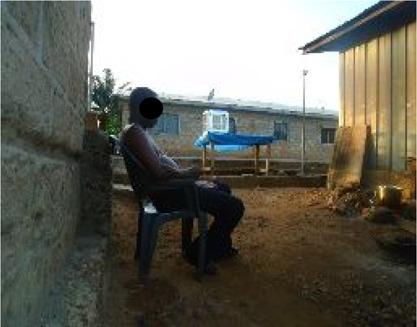
Accra	‘My father was a cook so we all learnt how to cook, I can prepare any food. I like Banku with Okra stew because it used to be the favourite food of my dad’ (Male, 50 years and above, lowest SES, A24).	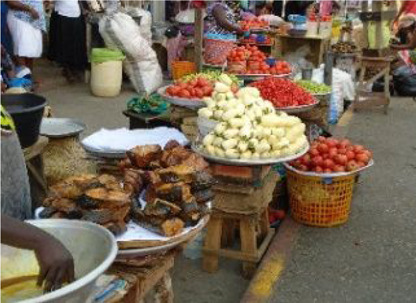
Nairobi	‘I love melon because it is my mum who influences me to eat melon because she loves melon a lot, most of the times you find that she buys melon a lot, like daily melons are in the house’ (Female, 19–49 years, lowest SES, N20).	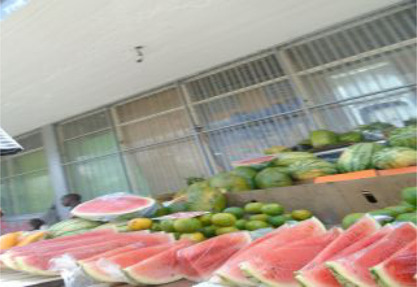
Accra	‘Since my childhood, she (mother) is the only woman who has been cooking for me to be well satisfied and she is the only woman who in this area cooks and makes me healthy… She influences me with the foods that she cooks, sometimes rice, egg stew, sometimes too rice and okra stew which makes me healthy’ (Male, 13–18 years, low to middle SES, A13).	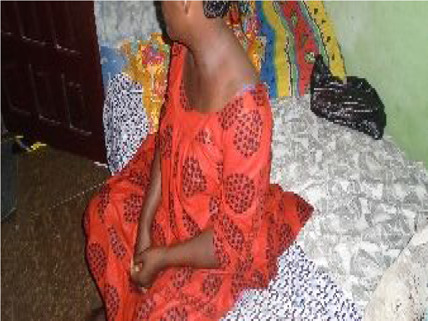
Ho	‘My mum and my dad, they take green pepper and lettuce. But I don’t like it. When she cooks it, I will not be able to remove them from the stew, so I must eat it like that. Also, sometimes, when she cooks jollof *(spicy west African rice dish),* she doesn’t always use meat, sometimes she uses fish. So, when you are eating the jollof, you are eating fish as well. I don’t like fish, if I was the one cooking, I would use meat’ (Female, 13–18 years, low-middle SES, H30).	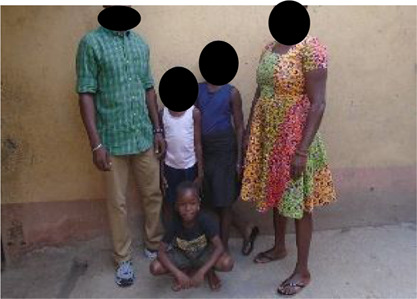
Nairobi	‘This is the food that our parents cook but I don’t like it so much I prefer other foods. It is the food that my mother makes me eat, I don’t like to eat it, this one for avocado, banana and ugali *(corn flour meal)’* (Male, 13–18 years, lowest SES, N47).	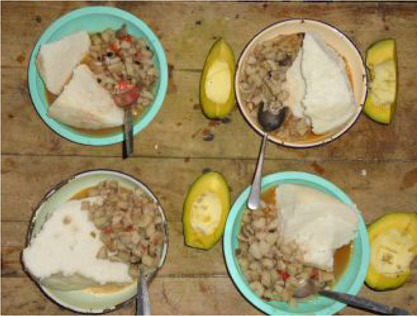
Accra	‘The nurse’s advice that we should be eating more frequently. We should not wait when we are hungry before we decide to eat. The eating interval should be short because the child in the womb eats from the mother so you have to eat frequently’ (Female, 19–49 years, low to middle SES, A50).	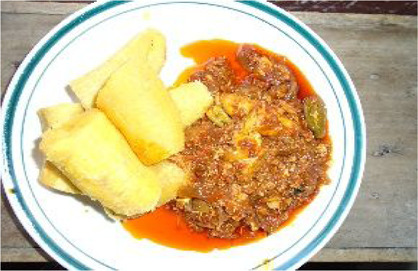
	‘These health workers (community health nurses) come around to advise us on how children should be fed and what to give them daily. But they don’t talk to us, the adults. It’s rather when you are sick and goes to the hospital that you are told what to eat and what not to eat’ (Male, 50 years and above, lowest SES, A24).	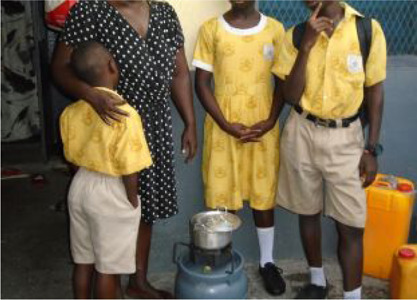
Nairobi	‘The doctor taught me if it is Ugali, the vegetables should be many and I should not exceed two pints of milk in the tea. That is the doctor’s advice so as to avoid cholesterol since cholesterol is what spoils your body’ (Female, 50 years and above, lowest SES, N12).	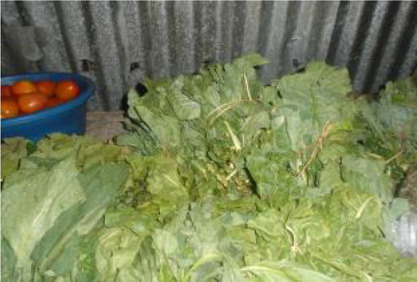

### Food vendors’ services and social qualities

In all three cities, the social qualities of local, mainly informal food vendors, largely influenced participants’ decisions on the places where they purchased food. Most participants preferred purchasing from food vendors who are friendly and hospitable, and avoided those who were impolite. Cleanliness of food vendors (referring to their appearance or how they dressed or smelt) and their food handling practices were key considerations by participants when making decisions on where to buy food. Participants in the three cities preferred food from vendors with whom they had established a good relationship, as it led them to trust the quality of foods sold. Food vendors providing credit services were also preferred by most participants, as they would always have access to food even when they did not have enough money. In Accra and Nairobi, provision of additional food services such as cutting /chopping vegetables and packaging were a consideration during food purchasing (Table 7).

**Table 7:** Narratives and photographs on the theme ‘food vendors’ services and social qualities’

Accra	‘Sometimes you get to the food seller and the food will be cold and when you ask her to heat it for you, she will insult on you. This doesn’t show respect and you leave and not go there again’ (Female, 13–18 years, lowest SES, A41).	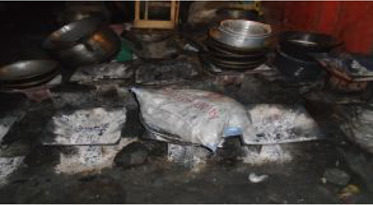
Ho	‘The way they talk to you, ‘Please, what are you buying?’ or ‘Please, thank you.’ You will be very happy then you will leave there glad. Even with how they will show their appreciation, you will want to go back and buy from there’ (Female, 13–18 years, lowest SES, H14).	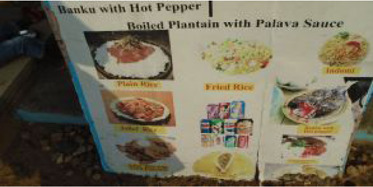
Nairobi	‘There is one that I buy from, the first day he/she talked to me nicely and that is the reason why I buy from there’ (Female, 19–49 years, low-middle SES, N4).	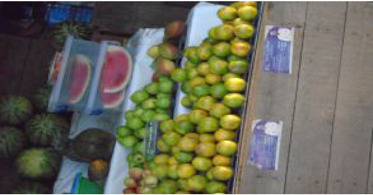
Accra	‘There was a time a woman was roasting plantain that was nice but the woman selling the food, she was dirty. When I looked at the fingers, it didn’t attract me… So I didn’t buy it anymore’ (Male, 50 years and above, low to middle SES, A25).	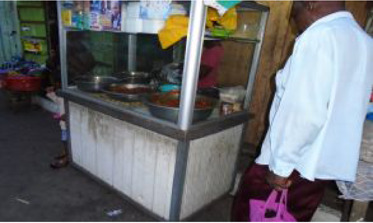
Ho	‘(I consider) how they take care of the surroundings and how they take care of the food, when you go and eat, because you will enjoy the food and also feel the sweetness in the food, you will go there again. This will even pull customers to them’ (Female, 19–49 years, low-middle SES, H23).	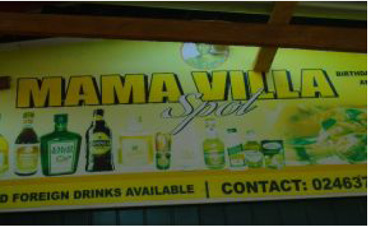
Nairobi	‘She makes sure that she has washed those (vegetables) for you in clean water, for me it’s about cleanliness if you see me going somewhere it’s because of cleanliness’ (Male, 50 years and above, lowest SES, N39).	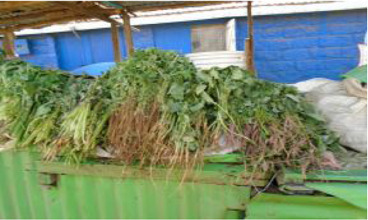
Accra	‘She is good to me because at times when I don’t have money, I go there and explain to her and she gives me food stuff to go and cook and when I get money I go back to pay her. This helps me a lot and makes my eating easy for me’ (Male, 50 years and above, lowest SES, A28).	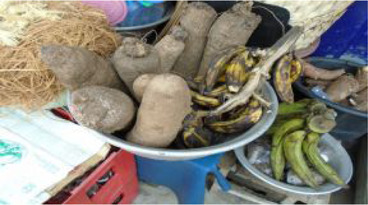
Ho	‘I know she does it for me, I don’t have any issue with her, and so I am able to get things on credit’ (Female, 19–49 years, low to middle SES, H27).	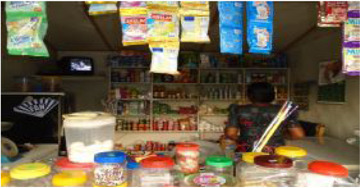
Nairobi	‘This shop belongs to my neighbour we have known each other for many years and we have brought our children up together you can see that girl I send her there I like there because if I do not have she can lend me’ (Male, 50 years and above, lowest SES, N12).	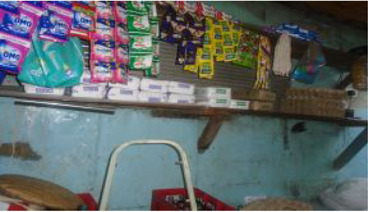
Accra	‘They do the grinding nicely for you and the millers always have a smile on their faces when I go there. On some occasions I may not have the amount they charge for grinding but they do not turn me away. They take the things I have and grind it for me’ (Female, 19–49 years, lowest SES, A38).	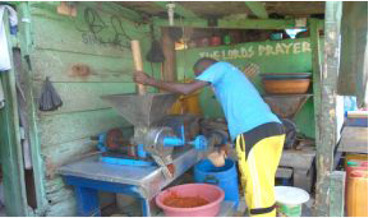
Nairobi	‘It influences my eating because the man selling there is, he knows how to cut, when you go to purchase cabbage, you can tell him the size you want, and he can cut big ones or small ones’ (Male, 19–49 years, low to middle SES, N28).	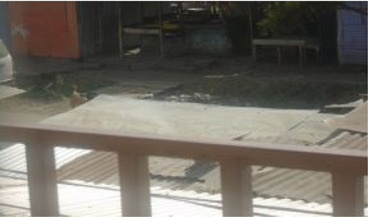

## Discussion

This study set out to explore the factors in the social food environment that influence dietary behaviour in urban cities, in Kenya and Ghana. The findings highlight the important role played by the social environment in shaping individual’s dietary behaviours.

Family plays a critical role throughout Africa and is seen as a source of identity and support^(25)^. The impact of family relations including parents, spouses, children, grandparents and siblings on individuals’ health behaviour and wellbeing is documented in other settings^(26)^. Family socialisation and habits were strong influencers of individual members’ food habits in disadvantaged communities in the United Kingdom^(27)^, and food selection for the family in urban Ethiopia^(28)^. Family members and household structure have also been shown to previously influence food choices and sourcing among Ghana’s urban poor communities^(29)^.

In this study, considerations for specific member’s food preferences were a major influencer on the household’s food purchase, preparation and consumption. For instance, women in their role as ‘mothers’ or ‘wives’ were seen as key influencers of family members’ dietary behaviour by incorporating the nutritional needs and preferences of their family members in food preparation and purchase decisions or by providing nutrition advice to their children. Similarly, women in Singapore reported to consider the health and nutrition needs of their children, and also the food preferences of various family members, especially their children and spouses, while making decisions on food purchase and preparation^(30)^. In Ethiopia, women acknowledged that they give in to their children’s food preferences as a way of encouraging them to eat^(28)^. Results from this study (published elsewhere) further indicated that family food preferences were also shaped by the family’s food access and availability^(23)^, which explains the preference for foods that most family members liked or could eat, which was seen as convenient, time and resource saving.

Younger participants acknowledged to have their dietary behaviour shaped by their parents’ choices on foods purchased, prepared and consumed by the family. Other studies indicate that parents influence their children’s behaviour through providing food, modelling dietary behaviour or encouraging specific dietary patterns^(31–33)^. This influence may also be through exerting authority in food provision in the household or negotiating with children on the foods prepared or available at home^(31)^, providing information on food selection and guiding them on good nutrition or preventing them from consuming hazardous food^(32,34)^. In Accra, it has been reported that mothers influence their children’s food behaviour by teaching them how to cook and choose the foods to eat^(29)^. Health professionals to a lesser extent also influenced individual health and dietary practices, through provision of information and advice, mainly to pregnant and lactating women. Our findings are similar to another study among young adults in Ghana’s Accra city which found that health professionals were among the key sources of nutrition information and were also perceived as the most credible sources of information^(35)^. In the smaller city of Ho in our study, this influence was found to be less common. This finding suggests that there is more opportunity for health professionals to influence healthy nutrition in urban populations, especially in smaller city contexts and to broaden nutrition information offered beyond pregnancy and the early years of life^(36)^.

Social relations and support plays an important role in health and diet behaviour. In this study, children and friends appeared to be an important factor among the older and younger participants respectively through provision of food and pleasant company during meals. Eating together as a family was also seen a time for family bonding and interactions. Similarly, in the USA, social support mediated the effect of nutrition interventions in improving dietary behaviour in middle aged and older adults^(37)^ while families that reported to eat their meals together exhibited a higher healthy index score^(38)^. A study in South Africa further concluded that individuals who experienced support from their friends and family in adopting a healthy diet were more likely to be motivated to identify healthy eating as their autonomous goals^(39)^.

The younger participants in our study reported enjoying the company of their friends, highlighting that it ‘felt good’ eating together and ‘sharing’ some foods, than eating alone. In the same vein, a qualitative study in Lima Peru identified peers as among the key influencers of adolescents dietary behaviour, through sharing of foods such as energy dense snacks and sweetened beverages^(34)^. In Indonesia, eating together at school was considered an important social activity in forming and maintaining friendships and peer groups^(40)^. A review by Stok et al.^(41)^ revealed that peer social norms influence food behaviour and that manipulation of these norms to promote certain food behaviours could yield significant beneficial results. Integrating social support in interventions targeting individual’s nutrition is therefore prudent. Interventions targeting adolescents and youth could also incorporate peer groups as avenues for delivering interventions to promote healthy dietary behaviour in these population groups.

Men in their role as ‘husbands’ or ‘fathers’ were sometimes referred to as providing supportive roles in food provision, purchase and preparation. This indicates that men may have ultimate influence on the food purchased and consumed in the household. A major difference, however, between the Kenya and Ghana cities was the influence that husband or male involvement had on family members’ dietary behaviour, through support with food preparation as reported in the data from Accra and Ho but not in Nairobi. The Nairobi findings are similar to those in previous studies in Uganda, Malawi and Ethiopia, where the role of men is depicted mainly as providing finances for food purchase^(28,42,43)^, with little or no involvement in decision making on foods prepared or consumed in the household. This could be because food preparation and purchase are traditionally viewed as the primary role of women in the African culture. A study in Malawi highlights the challenge of men feeling stigmatised if they are seen to be taking up ‘women’s’ traditional roles, such as food preparation^(43)^. The Malawi study however revealed a progressive shift in gender roles from the perceived traditional role of men as merely ‘financial’ food provision to actively supporting women in food preparation, sourcing and purchase, which aligns with situation observed in Ghana in the current study. Development and enforcement of government policies to encourage greater male involvement in food and nutrition issues as well as gender equality advocacy programmes were some of the strategies employed in central Malawi, to enhance men involvement in food and nutrition issues^(43)^, which could also be applied in other settings.

It was apparent in this study that food vendors have influence on individuals’ choices on food sourcing and provisioning. Food vendors play an important role in urban food systems, since a significant proportion of the food consumed by the urban poor is retailed by street vendors^(44)^. In a review by Pawel et al. (2012), characteristics of food sellers, including their friendliness and courteous service, were identified as determinants of the place of food purchase by consumers^(45)^. In our study, food safety, especially hygiene and sanitation in the food outlets, was raised as a concern by the study participants as discussed in a separate publication^(23)^ which may explain the preference for food vendors who appeared clean and prepared their foods hygienically. A Photovoice study with adolescents in urban Ethiopia also highlighted food vendors hygiene as a major consideration and influencer of their dietary behaviour^(19)^. Other studies document that consumers’ relationship with food suppliers influences their choice of food purchase; long-term relationships and food supplier’s reputation are among the key considerations of clients^(46)^. In Accra, consumers have been reported to be motivated by relationships with food vendors and good customer care practices when making food purchase decisions^(47)^. Urban low-income settings in Ghana and Kenya are prone to food insecurity^(44,48)^, and buying food through credit is a common coping strategy for food insecure households in urban settings^(49,50)^. In addition, results from this study also highlighted economic barriers to food access and overpricing of food products by some of the local food vendors as a potential hindrance to healthy eating^(23)^. This may explain the preference for food vendors who offer goods and services on credit in this study. In Ethiopia, buying food from food vendors who provide services such as packaging food in small and cheaper portions, and also credit services were reported as coping strategies for families experiencing food insecurity^(28)^. Developing, implementing and enforcing hygiene and safety regulations for food outlet owners were recommended by the participants in this study to ensure that food vendors maintained cleanliness as highlighted in a previous publication^(23)^. In addition, inclusion of food vendors in interventions aimed at improving populations’ dietary behaviour through provision of and increased access to healthier, hygienic and safer foods, will be prudent, given their influence on food purchase and dietary behaviour.

### Strengths and limitations

It is recommended that repeated group discussions are conducted throughout a Photovoice project to facilitate the community’s full engagement in the research. In our project however, only individual in-depth interviews were conducted due to logistic constraints of it being too difficult to find convenient times when a group of the urban low-income participants could come together frequently given other constraints on their time. We acknowledge this as a limitation, but we strived to ensure community engagement via separate community events. For example, a photography exhibition was held in each city to raise awareness of the drivers of unhealthy food consumption in the targeted low-income communities. The photography exhibitions served as a platform for community dialogue between study participants, the media and local government officers, during which, issues in their food environment and policy implications were discussed^(23)^. The qualitative study we conducted focused on understanding individual, social, physical and macro-level drivers of dietary behaviours. One paper combining all levels and describing the interactions between these four levels would have provided valuable information. However, we conducted 142 in-depth interviews across the three cities and gathered a large volume of data and photographs which provided a detailed, rich and comprehensive account of the drivers of dietary behaviours in the targeted African cities. As such, we wanted to describe in an in-depth manner those influences and pathways through which these factors may influence dietary behaviour and hence the choice to split the different components of the study into different papers^(23)^.

A key strength of this study is that it provides empirical findings from three cities in two African countries. Using the Photovoice methodology allowed participants to visually present social issues that influence their dietary behaviour. It also allowed active participation of respondents in the data collection, not only as mere respondents but playing an active role in identifying, capturing and describing the social influencers of their dietary behaviour that they perceive as important. The photographs taken by the participants allowed the research team to better appreciate the issues presented and hence facilitate richer discussions on these issues. This approach was enriching to the quality of the data collected and presented in this paper.

### Policy implications

In cognisance of the role that the family plays in influencing individual members’ dietary behaviours, interventions focusing on enhancing dietary behaviour at the individual level should consider and leverage the existing household and family structure, and their interconnectedness to be successful. In addition, friends and peers were common influencers of food consumption behaviour among younger participants. As such, peer groups may be considered as effective avenues for delivering interventions targeting adolescents. Food vendors influence food purchase behaviour. Empowering them to provide healthier and safer food options could also enhance healthier food sourcing, purchasing and consumption in African low-income urban communities. Healthcare workers influence nutrition knowledge, through provision of nutrition advice predominantly to pregnant and lactating mothers and mothers with young children, revealing a gap in the interaction with healthcare workers regarding diet for those not falling into these categories. The aspects of the social food environment highlighted in this study, how they influence dietary behaviour, and the population groups that they are most relevant to, should be considered when developing context and population-specific interventions to enhance healthier dietary behaviour.

## Supplementary material

For supplementary material accompanying this paper visit https://doi.org/10.1017/S1368980022002270. click here to view supplementary material
