# Supplementary material for: Community perceptions on the factors in the social food environment that influence dietary behaviour in cities of Kenya and Ghana: a Photovoice study
Source: Public Health Nutr. 2022 Oct 28;26(3):661–73. doi: 10.1017/S1368980022002270 (PMC9989710; doi:10.1017/S1368980022002270)
Supplement: Supplementary file 1 [file S1368980022002270sup.zip › S1368980022002270sup002.docx]

**Supplementary file 2: Photovoice Interview Guide**

**Presenting the photo voice exercise to participants**

Good morning/afternoon (name of participant), we are here to discuss the photo voice activity we told you about during the 24hr recall data collection a few weeks ago.

What is Photovoice?

A process of collecting information and expressing issues and concerns through photos. Photovoice asks participants to use photographs to record aspects of their lives and experiences.

In this project ***(DIETARY TRANSITIONS IN AFRICAN CITIES)***, we will be asking you to take some pictures of the things that make you choose the foods and drinks you consume at home or elsewhere (e.g. people you relate with, your environment, and any other things). We are doing this, so that we can help the formulation of policies that improve diets.

We will give you a digital camera which you will use to take photographs on some topics we have identified in a topic list below.

The pictures you will take and the discussion we shall have with you on your pictures, will help us understand how our family members, friends, neighbours, the places we stay in and the places we go, affect or influence the foods we eat and drink.

Furthermore, these pictures will help us to find ways that could make women and adolescent girls in Ghana eat and drink more healthily. To this end we would like to share some of the photographs that you and the other women in this project take in an exhibition later in the project so that people who make decisions about food and drink in this neighbourhood can have a better idea of the things that influence the foods you eat and drink. Your pictures can therefore be powerful in making change!

This Photovoice exercise will be in two sessions.

Today, we will take you through the picture list and how to use the camera. We will show you how to turn the camera on/off, how to snap the pictures and how to check if the pictures are on the camera or not. Then we will go through the picture list.

There are some few things to note:

**Photo Ethics and Safety**

- If you take a picture and the face of person shows, you will need to ask permission from the person before the picture can be used. We will give you a ‘Photo Release’ Form to use to get permission.
- Though we want you to take these pictures for us, please ensure you are safe anytime you go out to take pictures.
- You can go with another person to take the pictures.
- Don’t do anything you wouldn’t usually do.
- Don’t go anywhere you wouldn’t usually go.
- Please do not enter into a person’s private space to take your pictures.
- Avoid disclosure of embarrassing facts about individuals through your pictures.
- Avoid taking pictures of people in such a way that leaves a negative and inaccurate impression about that person.

In the second part, we will come after 7 -10 days for the camera, have the pictures printed and then come back for a discussion on the pictures. During the 7-10 days, we will check in to see how you are doing and check the progress of the picture taking, as well as answer any questions you have with regards to the picture list or the camera we gave you.

Let's start with showing you how to use the camera and after, we will go through the picture list. Please let us know if you do not understand or something is not clear. We will take you through it till everything is clear.

**PICTURE LIST**

Please try and take at least one picture that shows each of the 11 headings/topics below

1)  A place where you eat food and/or drink

2) Something that makes eating healthy difficult for you (for example, time; price of food/drink; home; work; school; neighbourhood/community; restaurants and fast food outlets; supermarkets; convenience and corner store).

3) Something that makes eating healthy easy for you (for example, time; price of food/drink; home; work; school; neighbourhood/community; restaurants and fast food outlets; supermarkets; convenience and corner store).

4) something that influences what you eat in your neighbourhood/community (for example, income/money; home; work; school; neighbourhood/community; restaurants and fast food outlets; supermarkets; convenience and corner store).

5) A person that influences your food or drink choice in your area (i.e. family; friends; peers; other)

​​

**INTERVIEW PROTOCOL**

1. Ask Participants to return with SD card after 7-10 days
2. Print photographs for interview
3. Match photo numbers with photo topics for data storage and identification on the day of interview.

Remind participant

- 1. In this project, we are trying to understand the things that make you choose the foods and drinks you consume at home or elsewhere (e.g. people you relate with, your environment, and any other things).
  2. We are doing this, so that we can help the formulation of policies that improve diets.

1. Start qualitative interview (see guide below) using pictures as focus for the interview

**Photovoice: Qualitative Interview Questions**

- Can you pick the most important picture to you please?
  1. Why have you chosen this picture over other pictures you took to include?
  2. Can you tell me about what this picture shows? *(note: try and get a thorough description of the photos and make sure the participant has covered why the picture is important to understanding food choices in their daily life).*
  3. If you had to tell one sentence to tell the story of this picture to a person looking at it in an exhibition what would it be?
- Which picture do you want to talk about next? *(note: use this prompt for all other pictures left to discuss)*
- For each picture:
  1. Can you tell me about what this picture shows? *(try and get a thorough description of the photos).*
  2. Can you tell me why this picture is important to understanding your food choices in your daily life? *(ask this only if this has not been covered in the description of the picture above).*
- If you could choose a photo to appear in an exhibit to tell a story about the food and drink environment in your community (good or bad things), which one would it be and why? *(note: If photo contains a face, check a photo release form exists, if not, choose another picture)*
- Can you describe any photos you would like to have taken to tell us about food choices in your neighbourhood/community that you were not able to take? What stopped you being able to take these?
- Ask participant to say (or write) a short caption/sentence that best describes what their picture is showing *(note: only ask this if the participant did not come up with a caption/one sentence description when discussing this particular photo earlier).*

1. Debrief - What is your general feeling about this activity?
   1. Did you learn anything from this activity e.g. about what influences consumption of food and drink in your community?
   2. What was your experience with learning to handle the camera?
   3. Did you feel safe handling the camera outside?
   4. Do you have any suggestions for improving the process?
