# Supplementary material for: Community perceptions on the factors in the social food environment that influence dietary behaviour in cities of Kenya and Ghana: a Photovoice study
Source: Public Health Nutr. 2022 Oct 28;26(3):661–73. doi: 10.1017/S1368980022002270 (PMC9989710; doi:10.1017/S1368980022002270)
Supplement: Supplementary file 1 [file S1368980022002270sup.zip › S1368980022002270sup003.docx]

**Supplementary file 1: Quota sampling plan**

This study was based on two sister projects: DFC and TACLED. The DFC project was only conducted in Ghana (Accra and Ho) whilst the TACLED project was conducted in both Ghana (Accra only) and Kenya (Nairobi). The sampling strategy for these two projects is presented below.

**Table 1a: Quota sampling plan for the Photovoice activity in Accra (DFC project)**

|  | | | | | | | | | |
| --- | --- | --- | --- | --- | --- | --- | --- | --- | --- |
| **SES**  **Reproductive**  **Life Course** | Lowest SES | | | | Low to middle SES | | | | **N** |
| 13-14y (not pregnant or  lactating) | Not in work or education | | In work or education | | Not in work or education | | In work or education | | 8 |
|  | BMI<25 | BMI≥25 | BMI<25 | BMI≥25 | BMI<25 | BMI≥25 | BMI<25 | BMI≥25 |  |
| 15-49y (not pregnant or lactating) | Not in work or education | | In work or education | | Not in work or education | | In work or education | | 8 |
|  | BMI<25 | BMI≥25 | BMI<25 | BMI≥25 | BMI<25 | BMI≥25 | BMI<25 | BMI≥25 |  |
| 15-49y (pregnant) | Not in work or education | | In work or education | | Not in work or education | | In work or education | | 8 |
|  | BMI<25 | BMI≥25 | BMI<25 | BMI≥25 | BMI<25 | BMI≥25 | BMI<25 | BMI≥25 |  |
| 15-49y (lactating) | Not in work or education | | In work or education | | Not in work or education | | In work or education | | 8 |
|  | BMI<25 | BMI≥25 | BMI<25 | BMI≥25 | BMI<25 | BMI≥25 | BMI<25 | BMI≥25 |  |
| **Total sample** | | | | | | | | | **n=32** |

**Table 1b: Quota sampling plan for the Photovoice activity in Ho (DFC project)**

|  | | | | | | | | | |
| --- | --- | --- | --- | --- | --- | --- | --- | --- | --- |
| **SES**  **Reproductive**  **Life Course** | Lowest SES | | | | Low to middle SES | | | | **N** |
| 13-14y (not pregnant or  lactating) | Not in work or education | | In work or education | | Not in work or education | | In work or education | | 8 |
|  | BMI<25 | BMI≥25 | BMI<25 | BMI≥25 | BMI<25 | BMI≥25 | BMI<25 | BMI≥25 |  |
| 15-49y (not pregnant or lactating) | Not in work or education | | In work or education | | Not in work or education | | In work or education | | 8 |
|  | BMI<25 | BMI≥25 | BMI<25 | BMI≥25 | BMI<25 | BMI≥25 | BMI<25 | BMI≥25 |  |
| 15-49y (pregnant) | Not in work or education | | In work or education | | Not in work or education | | In work or education | | 8 |
|  | BMI<25 | BMI≥25 | BMI<25 | BMI≥25 | BMI<25 | BMI≥25 | BMI<25 | BMI≥25 |  |
| 15-49y (lactating) | Not in work or education | | In work or education | | Not in work or education | | In work or education | | 8 |
|  | BMI<25 | BMI≥25 | BMI<25 | BMI≥25 | BMI<25 | BMI≥25 | BMI<25 | BMI≥25 |  |
| **Total sample** | | | | | | | | | **n=32** |

**Table 1c: Quota sampling plan for the Photovoice activity in Accra (TACLED project)**

|  | | | | | | | | | |
| --- | --- | --- | --- | --- | --- | --- | --- | --- | --- |
| SES  age group | Lowest SES | | | | Low to middle SES | | | | **N** |
| 13-18y | Not in work or education | | In work or education | | Not in work or education | | In work or education | | 8 males |
|  | BMI<25 | BMI≥25 | BMI<25 | BMI≥25 | BMI<25 | BMI≥25 | BMI<25 | BMI≥25 |  |
| 19-49y | Not in work or education | | In work or education | | Not in work or education | | In work or education | | 8 males |
|  | BMI<25 | BMI≥25 | BMI<25 | BMI≥25 | BMI<25 | BMI≥25 | BMI<25 | BMI≥25 |  |
| ≥50y | Not in work or education | | In work or education | | Not in work or education | | In work or education | | 8 males  8 females |
|  | BMI<25 | BMI≥25 | BMI<25 | BMI≥25 | BMI<25 | BMI≥25 | BMI<25 | BMI≥25 |  |
| 2 participants per cell | | | | | | | | | |
| **Total sample** (n=8 females; 24 males) | | | | | | | | | **n=32** |

**Table 1d: Quota sampling plan for the Photovoice activity in Nairobi (TACLED project)**

|  | | | | | | | | | |
| --- | --- | --- | --- | --- | --- | --- | --- | --- | --- |
| SES  age group | Lowest SES | | | | Low to middle SES | | | | **N** |
| 13-18y | Not in work or education | | In work or education | | Not in work or education | | In work or education | | 8 males 8females |
|  | BMI<25 | BMI≥25 | BMI<25 | BMI≥25 | BMI<25 | BMI≥25 | BMI<25 | BMI≥25 |  |
| 19-49y | Not in work or education | | In work or education | | Not in work or education | | In work or education | | 8 males  8 females |
|  | BMI<25 | BMI≥25 | BMI<25 | BMI≥25 | BMI<25 | BMI≥25 | BMI<25 | BMI≥25 |  |
| ≥50y | Not in work or education | | In work or education | | Not in work or education | | In work or education | | 8 males  8 females |
|  | BMI<25 | BMI≥25 | BMI<25 | BMI≥25 | BMI<25 | BMI≥25 | BMI<25 | BMI≥25 |  |
| 2 participants per cell | | | | | | | | | |
| **Total sample** (n=24 females; 24 males) | | | | | | | | | **n=48** |
